# Compartmentalized 3D Tissue Culture Arrays under Controlled Microfluidic Delivery

**DOI:** 10.1038/s41598-017-01944-5

**Published:** 2017-06-13

**Authors:** Burcu Gumuscu, Hugo J. Albers, Albert van den Berg, Jan C. T. Eijkel, Andries D. van der Meer

**Affiliations:** 1 BIOS Lab-on-a-Chip Group, MESA+ Institute for Nanotechnology, MIRA Institute for Biomedical Technology and Technical Medicine, University of Twente, 7500AE Enschede, The Netherlands; 2 Applied Stem Cell Technologies Group, MIRA Institute for Biomedical Technology and Technical Medicine, University of Twente, 7500AE Enschede, The Netherlands

## Abstract

We demonstrate an *in vitro* microfluidic cell culture platform that consists of periodic 3D hydrogel compartments with controllable shapes. The microchip is composed of approximately 500 discontinuous collagen gel compartments locally patterned in between PDMS pillars, separated by microfluidic channels. The typical volume of each compartment is 7.5 nanoliters. The compartmentalized design of the microchip and continuous fluid delivery enable long-term culturing of Caco-2 human intestine cells. We found that the cells started to spontaneously grow into 3D folds on day 3 of the culture. On day 8, Caco-2 cells were co-cultured for 36 hours under microfluidic perfusion with intestinal bacteria (*E. coli*) which did not overgrow in the system, and adhered to the Caco-2 cells without affecting cell viability. Continuous perfusion enabled the preliminary evaluation of drug effects by treating the co-culture of Caco-2 and *E. coli* with 34 µg ml^−1^ chloramphenicol during 36 hours, resulting in the death of the bacteria. Caco-2 cells were also cultured in different compartment geometries with large and small hydrogel interfaces, leading to differences in proliferation and cell spreading profile of Caco-2 cells. The presented approach of compartmentalized cell culture with facile microfluidic control can substantially increase the throughput of *in vitro* drug screening in the future.

## Introduction

In microfluidic platforms, compartmentalized culture models have been shown to provide spatio-temporally controlled microenvironments for monitoring intercellular activity and high-throughput handling of cells^1, 2^. For example, multiple replicates of a tissue construct can be simultaneously tested in microscale compartments, and various environmental physiological conditions can be screened at the same time in organ-on-chip platforms^3–5^.

Several techniques have been introduced previously for immobilizing cells on predesignated regions in microchips^6–16^. Micromolding methods have been used to encapsulate individual cells within microgel structures^6^. However, micromolding has a low consistency in the patterning success with respect to e.g. photolithography when it comes to the fabrication of periodic micron-sized arrays. Cell encapsulation has also been achieved by applying photolithography on photocrosslinkable synthetic polymers. This technique is widely used to create two-dimensional (2D)^7–9^ and three-dimensional (3D) cultures^10–12^, including cell-laden hydrogel microdroplets with precisely controlled geometries^13^. Despite offering high throughput, photolithography and microdroplet techniques require dedicated equipment, and are only compatible with custom-designed systems for photocrosslinkable polymers. As an alternative, microprinting has been used to create free-form patterned arrays of cell-laden materials^14^. For example, sphere-shaped functional tissues and organoids have been fabricated via bioprinters using natural and synthetic hydrogels^15^. In this technique, the extended surface area of sphere-shaped droplets containing the cells makes the droplets vulnerable to drying during the fabrication process. Other disadvantages of sphere-shaped tissue fabrication are limited resolution and the cell death possibility due to the shear forces in printing nozzles. Dielectrophoretic forces have also been utilized to concentrate cells into specific locations on microchips. This process however has advanced design and application requirements and, therefore, is not versatile^16^.

The aforementioned methods paved the way for high-throughput and scalable cell handling assays. Overall, these methods do not provide the ability to culture cells in a closed fluidic environment, which can be critical for mimicking physiologically relevant conditions, such as molecular transport and absorption directly from a continuous nutrient stream. Fluidic integration and fine fluidic control are essential if micropatterned cells are to be used for engineering organs-on-chips^17, 18^. This requirement has been recently addressed by the development of a ‘phaseguide’ technique, which can be used to pattern hydrogels and cells in microfluidic systems^19, 20^. The technique was recently used to manufacture a 3D co-culture of two different cell types embedded in adjacent lanes of gels, and is widely used in organ-on-chip applications^21^. The commercial platforms based on phaseguides only offer limited control over fluid flow, because flow control relies on altering hydrostatic pressures by adjusting fluid column heights. Active fluid control would require individual fluidic connections and tubing running to each of the parallel compartments, which would lead to very large experimental set-ups when addressing hundreds of 3D cultures in a high-throughput platform^21^.

Previously, our group has reported *in situ* fabrication of large arrays of periodic hydrogel compartments in a glass microchip by capillary pinning of liquids in microcompartments^22^. The capillary pinning method would be ideal for micropatterning of cells in 3D compartments since it allows for patterning large arrays of hydrogels directly inside a chip, making it thereby compatible with microfluidic flow control. In this study, we show the feasibility of this approach by applying the capillary pinning technique to fabricate approximately 500 periodic cell-laden hydrogel compartments in a single microchip made of PDMS. Well-controlled fluid flow in the microchip allowed us to culture human intestine epithelial cells (Caco-2) in the microchip^23, 24^ as well as to screen the glucose consumption rate of the cells. Long-term co-culturing of an intestinal bacteria (*E. coli*) and Caco-2 cells was also achieved in the microchip.

## Results and Discussion

### Cell filling and culturing

**Figure 1:**
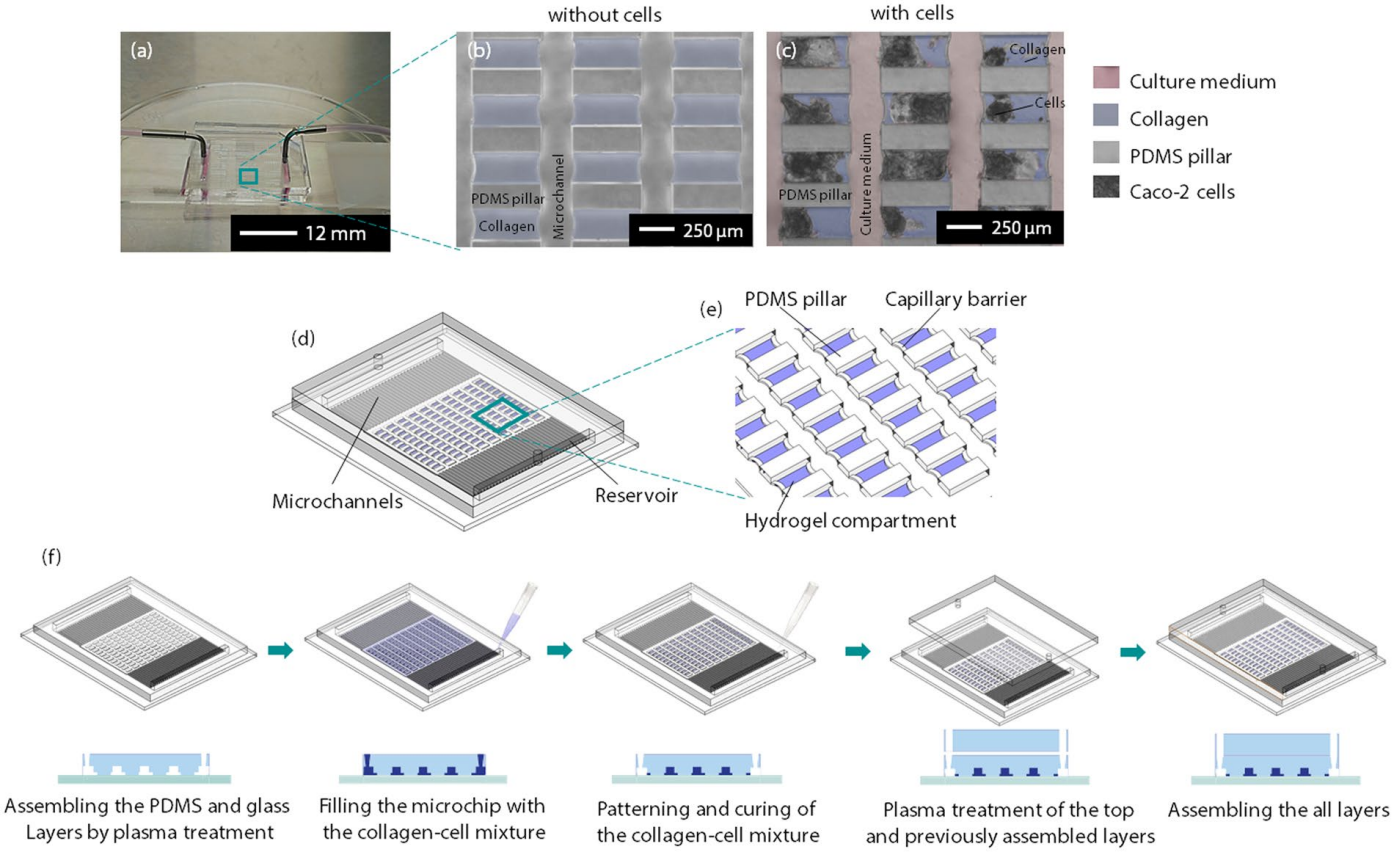
Overview of microfluidic chip design and the method of patterning hydrogels by capillary pinning. (**a**) A photograph of the microchip with attached tubing. (**b**) A zoomed in optical microscopy image of the PDMS pillar array (grey), hydrogel compartments (blue structures between glass pillars). (**c**) The pillar array with Caco-2 cells (on culture day 8), and culture media filled channels (pink). Microchannels and hydrogel compartments were pseudo-colored based on grey-scale differences. (**d**) Schematic isometric view of the microchip with hydrogel patterns. (**e**) A zoomed-in schematic illustration of the capillary barriers, PDMS pillars, and hydrogel compartments. The height of capillary barriers is 1/4 of the microchannel height. (**f**) Sequential steps of chip assembly and hydrogel patterning. The microchip was fabricated using standard soft lithography techniques.

The microfluidic cell culture platform was developed to enable long-term cell culturing in periodic 3D compartments under controlled fluid flow. To achieve that, a collagen pre-gel and Caco-2 cell mixture was patterned in the microchip using capillary line pinning. The patterning success by capillary pinning was evaluated on a passed or failed basis. Hydrogel compartments covering the entire gap between PDMS pillars were counted as passed. All other configurations were counted as failed. We found a patterning success rate of 95.0 ± 7.6% (mean ± standard deviation, n = 15), based on our analysis on five representative images that were collected from random spots in the microchips. The patterning success rate is in accordance with previous reports^22^. Figure 1b and c illustrate the top-view phase contrast microscopy images of the channel structure and patterned hydrogel compartments with and without Caco-2 cells.

### Culture conditions

Concentration of the collagen mixture and seeding density of the cells were optimized for maximizing cell survival and facilitating the formation of 3D structures. Four different collagen concentrations ranging from 0.3% to 0.45% (w/v) with 0.05% (w/v) increments were tested in the microchip. Caco-2 cells did not proliferate at concentrations higher than 0.3% (w/v) collagen (Supplementary Fig. S1a–d). In the preliminary experiments, four different cell concentrations in a range between 1.7 · 10^6^ and 1.3 · 10^7^ cells ml^−1^ were also tested. Cell concentrations lower than 6.7 · 10^6^ cells ml^−1^ resulted in no growth in the cell population. Higher initial cell populations made it difficult to control the patterning process. Therefore, a concentration of 6.7 · 10^6^ cells ml^−1^ was used in all of the experiments reported in this study.

### Glucose diffusion and flow velocity distributions in the microchip

The computational COMSOL model results showed very similar velocity fields when using the laminar and creeping flow modules (laminar flow displayed in Supplementary Fig. S2a). They also showed that the velocity in the outer microchannels was 30% lower than in the center microchannel, due to path length differences resulting in a different hydrodynamic resistance. Flow velocity distribution could be exploited or made uniform by varying channel widths in future designs.

The simulations furthermore showed that the expected glucose concentration distribution over the microchip is almost uniform, also in the presence of metabolically active cells (Supplementary Fig. S2b).

### Morphology changes at long-term cultures under static and fluidic conditions

**Figure 2:**
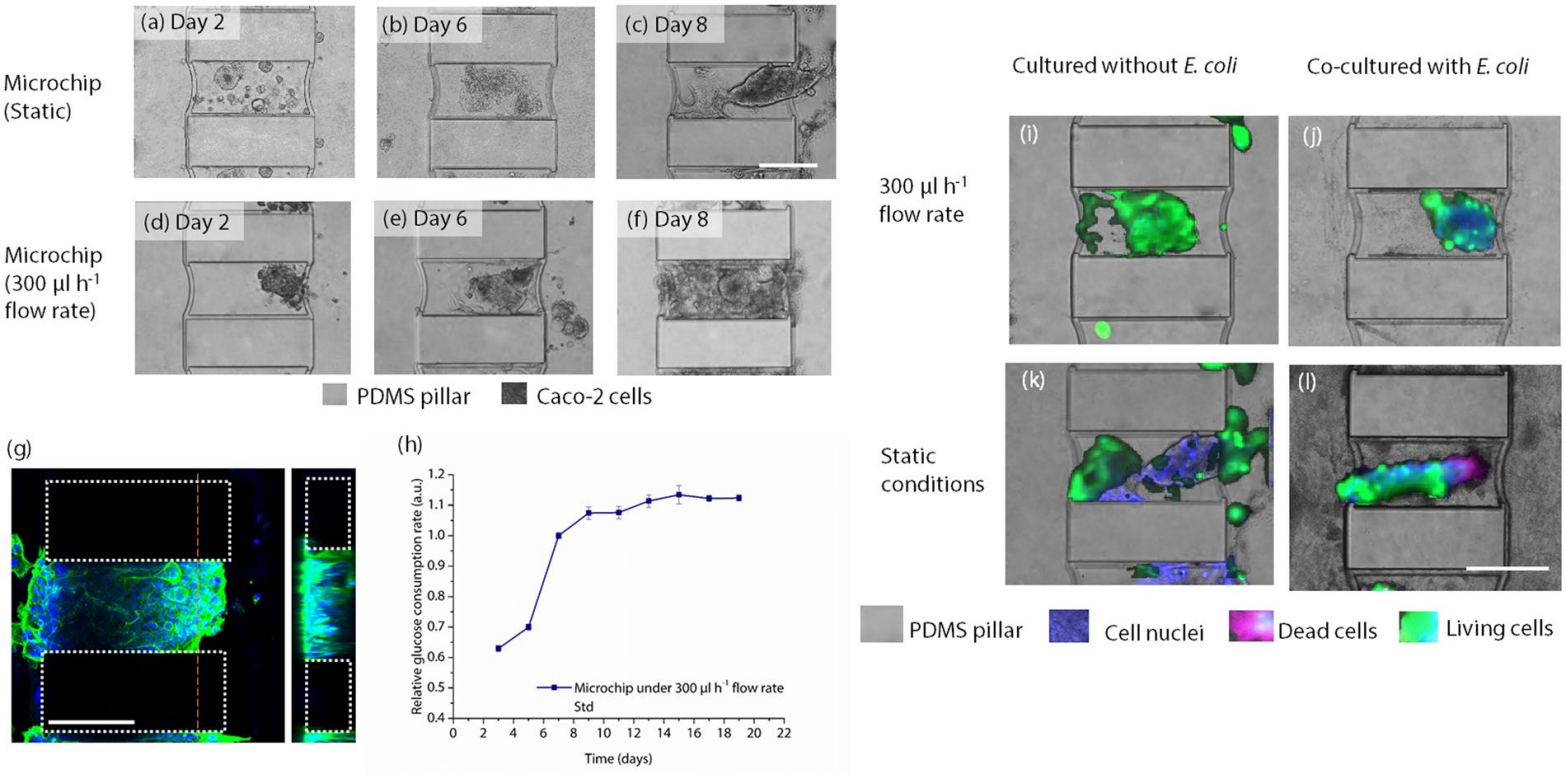
Left: Top-view phase contrast images of Caco-2 cells in the microchip in different days of cell culture. The results are shown for the microchips operated (**a–c**) under static conditions and (**d–f**) under 300 µl h^−1^ flow rate. (**g**) Confocal microscopy image of the Caco-2 cells grown under 300 µl h^−1^ flow rate on day 8. Dashed lines denote pillar boundaries, red dashed line shows location of y-z cross-section (inset right) (**h**) Glucose consumption rate of Caco-2 cells cultured inside the microchip over 21 days. Right: Top-view phase contrast microscopy images of live/dead assay bacteria co-culture operated (**i**) under 300 µl h^−1^ flow rate without *E. coli* cells, (**j**) under 300 µl h 1^−1^ flow rate with *E. coli* cells, (**k**) without fluid flow and without *E. coli* cells (**l**) without fluid flow with *E. coli* cells. The nuclei of Caco-2 cells were stained with DAPI (blue). Alive Caco-2 cells are shown in green and dead Caco-2 cells are shown in red colors. In (**j** and **l**) the dark cloudy appearance in the microchannels and the compartments is caused by *E. coli* colonies. All scale bars are 250 µm.

The cells were cultured under static and fluidic conditions during 14 days in microchips. Supplementary Fig. S3a–j and Fig. 2a–g provide the evidence for the morphological changes of the cells in this period.

When no flow was present in the microchannels, Caco-2 cells spread, however stayed in the hydrogel patch in the first 6 days. The cells tend to stay close to each other by forming clumps in the hydrogel compartment. On day 8, the cells started migrating towards the microchannels (Fig. 2c). However, cellular density, which was determined by the number of cell nuclei, was found to be low in the microchannels when compared to the hydrogel compartments on day 8. Cellular protrusions seem to be localized more in the microchannels with regards to the hydrogel compartments.

At 30 µl h^−1^ flow rate, the cells were often observed to localize next to the capillary barriers. After 21 days of culture, the cells did not occupy the microchannels in contrast to the higher flow rates, and cell proliferation was not observed in all cell-laden compartments. We interpret this result as an indicator of insufficient flow rate to supply nutrients to the cells (Supplementary Fig. S3a–e and Supplementary Fig. S2b)^25^.

**Figure 3:**
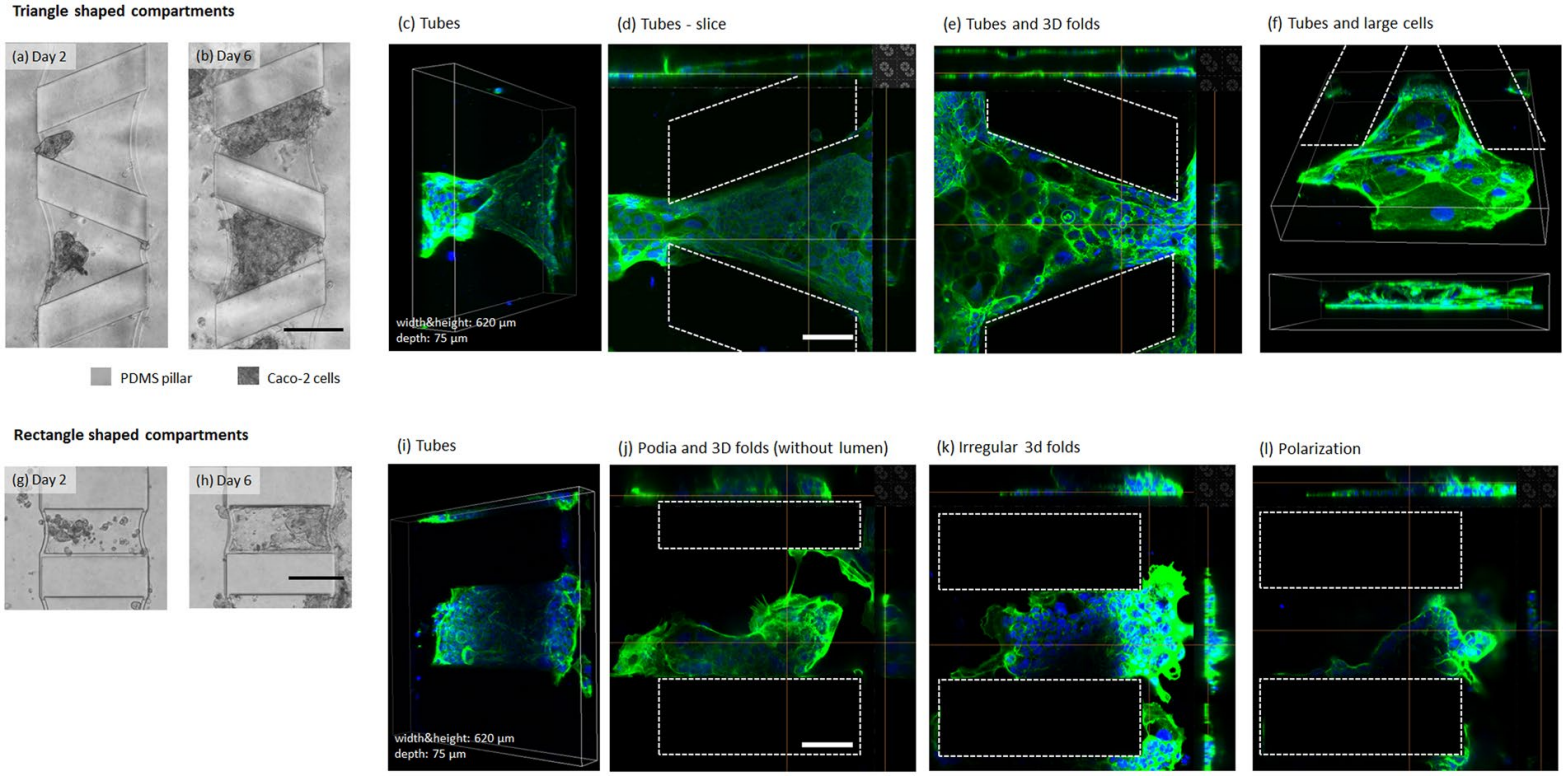
(**a**,**b,g,h**) Top-view phase contrast microscopy images of Caco-2 cells cultured under 300 µl h^−1^ flow rate in compartments with various sizes and shapes. (**a,b**) Triangular compartments and (**g,h**) rectangular compartments on days 2 and 6. Scale bars are 250 µm. (**c–f, i–l**) Top and 3D views of confocal microscopy images. Caco-2 cells cultured in (**c–f**) triangular shaped and (**i–l**) rectangular shaped compartment designs under 300 µl h^−1^ flow rate of culture medium. DAPI and GFP represent cell nuclei and actin filaments, respectively. Cells forming tubes, irregular 3D folds, and podia are shown. Scale bars are 125 µm.

When culture medium was perfused through the microchannels at 300 μl h^−1^ flow velocity, Caco-2 cells would form 3D structures in the hydrogel compartments on day 3 (Supplementary Fig. S3f). We observed that most of the compartments (~90%) were fully occupied by Caco-2 cells on day 8 of the culture. Between days 8 and 10, we observed that Caco-2 cells started to spread to the microchannels (Supplementary Fig. S3h). After day 15, the cells over-proliferated in the microchip and occupied the surrounding walls of the microchannels together with the space in hydrogel compartments (Supplementary Fig. S3g,h). Immunofluorescence microscopy experiments using labels directed against the F-actin filaments confirmed that Caco-2 cells formed confluent polygonal epithelial monolayers throughout the microchip on day 15 day of the culturing period (Supplementary Fig. S3j). The observed growth and migration of cells outside of the compartments after day 8 is unwanted, because this means the individual microcompartments are no longer independent culture environments, but instead are all connected. In the future, this may be prevented by first applying cell-repellent materials like polyethylene glycol by, i.e., microcontact printing^26^ or photopatterning^27^, before assembling the final device. In summary, phase contrast microscopy images show that application of the high fluid flow rate (300 µl h^−1^) accelerated cell proliferation and migration, as well as the formation of 3D structures. Formation of 3D structures was also confirmed by confocal microscopy on day 8 of the culture (Fig. 2g and Supplementary Movie S1). The majority of the cells covered the compartment walls in monolayers and eventually formed tubular structures with or without 3D folds inside as shown in confocal images in Fig. 3i–l. Signs of polarized 3D cell clusters with a continuous sub-apical F-actin signal were also observed in some confocal images (Fig. 3l and Supplementary Fig. S4).

Interestingly, 3D structures appeared under both static and fluid flow conditions after 6 days of culturing. The formation of 3D structures as well as cell proliferation rates were higher in the microchip perfused with culture medium, as seen in Fig. 2a–f. The timing of 3D structure formation in this study is consistent with the villi-like structure formation observed by the *in vitro* study of Kim *et al*.^23^ and several *in vivo* studies^28, 29^.

### Glucose consumption

To demonstrate that it is possible to analyze samples from the outflow of the chips, glucose consumption over 21 days was analyzed in outflow samples by using UV-Vis spectroscopy. For this study, the microchip was perfused at a flow rate of 300 µl h^−1^. The results shown in Fig. 2h reveal that the cells continuously consumed glucose with gradually increasing amounts in the first 10 days of the culture in the microchips. Glucose consumption in the chip stabilized after day 10, which corresponds to the phase where microchannels were covered with confluent monolayers of Caco-2 (Supplementary Fig. S3j). The qualitative correlation between glucose consumption and cell growth patterns indicates that the analysis of outflow samples from the chip provides information about the state of the cells cultured inside the chip. This result demonstrates that on-line monitoring of cell biological parameters is possible by continuous sampling from the chip, because on-line monitoring is a direct result of the active flow control of the system.

### Bacteria co-culture

Bacteria and human intestine epithelial cells were co-cultured in the microchip in both long-term and short-term experiments to determine the capability of our microchip system to support relevant co-cultures.

Figure 2i–l show representative images from the live/dead staining of the culture with calcein-AM and ethidium homodimer-1, respectively. Caco-2 cells showed no indication of cell death (no red colored cells were observed in Fig. 2j) when the culture was treated at 300 µl h^−1^ flow rate with a suspension of *E. coli* cells (1.9 · 10^7^ cells ml^−1^) for 36 h. The viability of Caco-2 cells was observed to be similar to a system under the same conditions without *E. coli* cells (Fig. 2i). When Caco-2 cells are co-cultured with bacteria under static conditions, approximately 30% of Caco-2 cells were dead after 36 h, as observed in live/dead staining images (Fig. 2l). When Caco-2 cells are cultured under static conditions only, almost 100% of Caco-2 cells remained alive (Fig. 2k). These results demonstrate that the microfluidic perfusability of the microchip effectively enhanced cell viability when micropatterned Caco-2 were co-cultured with *E. coli* cells. The fluidic environment prevented unrestrained over-proliferation of the bacteria in the microchip while Caco-2 cells remained accessible by the bacterial cells in the fluidic culture. In Fig. 2l, the cloudy regions in both the hydrogel compartments and microchannels—where no fluid flow was present—indicate unrestrained bacteria proliferation under static conditions. Contrarily, when 300 µl h^−1^ flow rate was applied (Fig. 2j), the cloudy regions were not observed in microchannels while they were present in the hydrogel compartments in the microchip.

In a short-term study, bacterial adhesion to Caco-2 cells was observed by injecting recombinant GFP-expressing *E. coli* cells into the microchip for 1.5 h (Supplementary Fig. S5). During the antibiotic-free culture media treatment, *E. coli* cells adherent to Caco-2 cells remained in the hydrogel compartments while non-adherent cells were washed out with the fluid flow. To illustrate its potential use for drug screening, we then treated the chip with chloramphenicol, a drug that has known activity against *E. coli* ^30^. Chloramphenicol is a broad-spectrum antibiotic that inhibits bacterial protein synthesis, resulting in bacteriostatic activity. Due to unwanted side-effects and growing bacterial resistance, the drug is hardly ever used in the clinic anymore^31^. However, in recent years, there is renewed interest in chloramphenicol as a tool for treating infections by bacteria that display multi-drug resistance^31, 32^. The minimum inhibitory concentration (MIC) of chloramphenicol for *E. coli* in standardized assays is 4 µg/ml^33^ and the minimum bactericidal concentration (MBC) is 250 µg/ml^34^. Typical serum concentrations in the clinic are in the range of 10–20 µg/ml^35^. Chloramphenicol is also a popular antibiotic for mammalian cell culture applications; as an additive for cell culture media, it is typically used at concentrations of 5 to 50 µg/ml^36^. When we used chloramphenicol at a typical concentration of 34 µg/ml, bacterial proliferation was not observed in the microchip after the incubation with the drug for 36 h. However, the adherent bacterial cells remained in the compartments as seen in Supplementary Fig. S5. This observation is in agreement with the fact that the drug is mostly bacteriostatic and not bactericidal at the used concentration.

Together, these findings demonstrate that the controllable fluidic perfusion in the microchip enables preliminary screening of drug effects in co-cultures of Caco-2 and bacteria.

### Effect of hydrogel compartment geometry on cell distribution

The fabrication method of the microfluidic chips enables flexible design of microcompartments of various sizes and shapes. Figure 3a,b,g,h and Supplementary Fig. S6 demonstrate the cell distribution in differently shaped compartments with smaller or larger hydrogel-culture media interfaces. Under static conditions, Caco-2 cells in the compartments with a large interface (Supplementary Fig. S6a,b) spread more evenly over the compartments probably due to the increased hydrogel interface, and therefore increased surface area in the hydrogel compartment. The morphology of the cell clusters seemed to be more elongated when compared to the round-shaped cell clusters in smaller hydrogel compartments (Supplementary Fig. S6c,d). We also patterned cells in microchips with triangular-shaped hydrogel compartments and compared the 3D cell distribution patterns with those in rectangular-shaped hydrogel compartments. Results shown in Fig. 3a,b,g,h suggest that in both geometries, cells initially localize to the regions close to the microchannels, probably due to the nutrient transport, and over time fill up the full compartments. The different cell distribution patterns that we observe in hydrogel compartments with different geometries could be due to many different reasons, like site-specific cell proliferation, cell death, directional migration, or cell clustering due to local hydrogel density or mechanics. Future studies will have to provide a more in-depth analysis of the underlying mechanisms.

Based on observations using the confocal microscope in Fig. 3, Caco-2 cells showed five distinct behaviors in the hydrogel compartments after 8 days of culturing: cells formed (1) tubular structures by covering both top, bottom, and side channels (Fig. 3c,d,i), (2) tubular structures with 3D folds inside, resembling a lumen structure (Fig. 3e,f), (3) polarized structures where nuclei lined up and remained in the middle of the cytoskeleton (Fig. 3l), (4) irregular 3D folds where no lumen was observed (Fig. 3k), and (5) podia (Fig. 3j). Formation of these structures was observed in both triangular and rectangular compartments.

## Conclusion

In this work, we have developed a new approach to build *in vitro* cell culture platforms for tissue mimicry using periodic 3D cell-laden hydrogel compartments inside closed fluidic microchips. The design concept is based on selectively trapping mixtures of collagen pre-gel and cells in compartments via capillary line pinning. The architecture of the microchip and the ability for continuous fluid delivery enabled long-term and in-parallel culturing of human intestinal cells (Caco-2) that spontaneously grew into 3D folds on day 3 of cell culturing. Confocal images suggest that cells tend to form five distinct structures in the microchannels, including tubes with lumen, tube structures with lumen and 3D folds inside, 3D folds with polarized cells, irregular 3D folds with no lumen, and cells forming podia. We also co-cultured Caco-2 cells with an intestinal bacterium *(E. coli)* to show bacteria-cell attachment and the viability of Caco-2 cells under fluidic conditions. *E. coli* bacteria adhered to Caco-2 cells after 36 h of incubation without affecting the viability of Caco-2 cells, showing cell-bacteria attachment in the microchip as well as mimicking of intestinal integrity in terms of bacteria-cell interactions.

3D spheroid and organoid tissue culture models are becoming increasingly popular in biomedical science and drug screening^37–39^. One of the most high-profile examples is the *in vitro* culture of 3D gut organoids from single adult stem cells^40^. Even though gut organoids have been highly successful as tools in medical biology^41^, drug screening^42^, and tissue engineering^43^, gut organoid cultures have shortcomings that are representative for most spheroid and organoid models. For example, they display size heterogeneity, their overall tissue shape is limited to folded spheres, and co-cultures with bacteria can only be performed for short periods of time due to bacterial overgrowth^44^. The introduction of the parallelized microculture platform and the proof-of-concept data that we provide based on 3D culture of Caco-2 intestinal epithelial cells, demonstrate that our approach may be used in future studies to address some of the aforementioned shortcomings of spheroid and organoid culture models in general, and of gut organoid culture in particular.

The fluidic control of the compartmentalized organ-on-chip devices was addressed in this study to enable on-demand manipulation of the outer cell microenvironment in well-defined structures. In our future work, several features of the microchip can be altered or added in order to use this approach for effectively mimicking the complex functions of different tissues, including response of the Caco-2 cells to the bacteria-cell attachment and application of different drugs and in the molecular level. Replacing the buffer reservoirs with single microchannels would for example make a more physiologically realistic *in vitro* model. In this way, the application of fluids with different compositions in each side of the compartments will be enabled. Finally, the fluidic control and active perfusion of our system will be useful in dealing with the well-known issue of drug absorption in PDMS-based systems. For some compounds, absorption to PDMS might lead to 85% to even 99% reduction of free compound in microfluidic systems in which the drug is statically incubated^45, 46^. Only by actively perfusing a system with the intended concentration for multiple hours will the intended concentrations be reached by saturation of the PDMS.

Our approach offers great promise for building next-generation organotypic *in vitro* platforms by enabling high-throughput culturing in a microfluidic environment, where approximately 500 hydrogel compartments can be easily fabricated to serve as bioreactors. In addition, this approach has the potential to be used for creating separate 3D microenvironments, where a gradient of different metabolites can be applied to study tissue functions, drug screening, and perhaps organ-on-chip assemblies. The microchip can be an alternative to the bio-microreactor systems, as it enables high-throughput measurements by the multiple microchannels designed in parallel to each other.

## Methods

### Microchip fabrication

The microchip was fabricated from polydimethylsiloxane (PDMS, Dow Corning) polymer using standard soft lithography techniques. The microchip consisted of one glass and two PDMS layers. The upper PDMS layer contained one inlet and one outlet while the lower PDMS layer contained pillars, capillary barriers, microchannels, and buffer reservoirs. The glass layer (microscope slide) was directly bonded to the lower PDMS layer. An assembled microchip is shown in Fig. 1a and d, consisting of a 14.5 mm by 9.5 mm rectangular chamber, connected to the buffer reservoirs via microchannels (200 µm × 5 mm with 200 µm periodicity) on upper and lower sides (Fig. 1b and e). The microchannels ensure the distribution of flow over the culture chamber (Fig. 1d and Fig. S1). An array of rectangular pillars and capillary barriers placed in the culture chamber served as a mechanical support for the fabrication of periodic hydrogel patches. In one microchip design, pillars and hydrogel compartments were of the same dimensions: 200 × 500 µm (width × breadth). In a second design, the hydrogel compartments were 600 × 500 µm while the pillar dimensions remained the same. In a third design, the PDMS pillars were 600 × 500 µm while they were placed with a zigzag pattern at angles of 20°, generating triangular hydrogel compartments. The microchannel height was 75 µm, while the capillary barrier height was 67.5 µm. The total inner volume of the microchip was approximately 40 µl.

The lower PDMS layer was prepared by casting a prepolymer (10:1 w/w ratio of PDMS to curing agent) on an SU-8 master mold that was fabricated by photolithography in the MESA + cleanroom facility at the University of Twente, The Netherlands. The process flow is shown in Fig. 1f. The SU-8 master contained the negative pattern of the microchip design and consisted of two SU-8 layers (Microchem). The first layer contained microstructures for the pillars and microchannels, while the second layer contained microstructures for the pillars, microchannels, and capillary barriers. The height of the first layer was 67.5 µm and that of the second layers was 7.5 µm, which were measured using a Dektak surface profiler (Bruker, Germany). After curing the prepolymer at 60 °C for 3 h, the PDMS layer was peeled off from the SU-8 master. The rectangular buffer reservoirs were then cut using a blade. The patterned surface of the lower PDMS layer was treated with oxygen plasma at 500 mTorr for 45 seconds using a Harrick Plasma Cleaner, USA, and immediately bonded with an oxygen plasma treated glass layer. The buffer inlet and outlet were opened using a hole puncher with 1.5 mm diameter in the upper PDMS layer, which was only bonded to the lower PDMS layer after hydrogel patterning. The microchip was used immediately after preparation. The tubing and the microfluidic connectors that were used in the experiments were sterilized by rinsing with 70% (v/v) ethanol and 1× PBS (phosphate buffered saline, Sigma Aldrich) solutions.

### Hydrogel patterning

A cell-containing collagen mixture was prepared by mixing 1 M NaOH (1.38% (v/v), sterilized, Sigma Aldrich), Dulbecco’s Modified Eagle Medium (DMEM) high glucose Glutamax medium (36% (v/v), ThermoFisher Scientific) suspended with Caco-2 cells (6.7 · 10^6^ cells ml^−1^), Collagen (0.3% (v/v), Trevigen Rat Tail Collagen type I), and deionized water (sterilized)^47^.

The patterning process occurs via capillary action and it is affected negatively by the hydrophobic recovery of PDMS after the oxygen plasma treatment. For this reason, the hydrogel-cell mixture was injected into the microchip immediately after the assembly. The microchip was completely filled with 40 µl of the mixture and the excess mixture in the main microchannels was removed using a Pasteur pipette connected to a vacuum pump. In this stage, the collagen mixture only remained between the pillars and capillary barriers due to the capillary pinning process^12^. No air bubbles were trapped during the entire patterning process as described in our earlier work on the design and working principles of capillary barriers^12^. After the patterning process, the microchip was placed in the incubator for 1 h to allow the collagen mixture gelation.

### Cell culture

Human Caco-2 intestinal epithelial cells (ATCC HTB-37 Caco-2 cell line) were cultured as a monolayer in tissue culture-treated polystyrene cell culture flasks (Nunc) in DMEM high glucose Glutamax medium (Gibco) supplemented with 20% (v/v) Fetal Bovine Serum (FBS, Gibco), 100 units ml^−1^ penicillin (Gibco), and 100 units ml^−1^ streptomycin (Gibco) using an incubator set at 37 °C and 5% CO_2_ (Binder). The culture medium with this composition was used in all experiments unless stated otherwise. During the culturing process, the culture medium was refreshed every 3 days until the cells reached 80% confluency. Caco-2 cells were then harvested using trypsin/EDTA solution (0.05%, v/v, Gibco) and suspended with DMEM Glutamax medium with a final cell concentration of 6.7 · 10^6^ cells ml^−1^ prior to the patterning of collagen mixture. At this cell concentration, aggregation or superposition of cells was not observed in hydrogel patches. After the patterning process, the microchip was incubated for 1 hour and culture media was subsequently pumped into the microchip at a constant flow rate (30 or 300 µl h^−1^) using a Harvard PhD 2000 syringe pump.

The medium in the static microchip was refreshed every three days. All experiments with cells in chips and Transwells were carried out inside an incubator set at 37 °C and 5% CO_2_.

### Computational modeling of fluid flow and glucose diffusion

Flow velocity and glucose diffusion in the microchip were simulated using COMSOL Multiphysics software. Creeping flow, laminar flow and transport of diluted species modules were used in simulations. A volumetric flow of 300 µl h^−1^, a glucose concentration of 25 mmol l^−1^ were used in the simulations^48^. The creeping flow module was operated by applying steady flow, incompressibility, negligible inertial forces and shallow channel approximation conditions. For the laminar flow, we applied steady flow, incompressibility, and a shallow channel approximation. The transport of diluted species was coupled with the calculated convection from either the laminar or the creeping flow module. The diffusion rate of glucose in water is D_cW_ = 6.8 · 10^−10^ m^2^ s^−1^ ^49^, the diffusion rate of glucose in the hydrogel patch is D_cH_ = 1.441 · 10^−10^ m^2^ s^−1^ ^50^. For the consumption rate of glucose, the glucose uptake of 500 Caco-2 cells per hydrogel patch was estimated to be −20 · 10^−2^ mol m^−3^ s^−1^ ^50^.

### Glucose measurement

We measured the glucose consumption over 14 and 21 days of the cell culture in the microchip. Culture medium was collected from the outlet every two days. Collected samples were then transferred to a 96-well plate to quantify the glucose concentration using a Multiskan GO (ThermoFisher Scientific, USA) microplate reader at 278 nm^18^. Unconditioned culture medium was used as the blank, and a calibration curve of glucose was obtained by measuring different concentrations of the cell culture media containing 25 mM glucose. The resulting glucose concentrations were in the range between 2.5 mM and 25 mM (Supplementary Fig. S7). The obtained data points were normalized to the glucose consumption rate on day 7 of the culture in the microchip.

### Bacteria co-culture

*Escherichia coli* cells were utilized to study cell-bacteria attachment and interactions. *E. coli* cells (ATCC, USA) were incubated in 10 ml sterile Luria Bertani (LB) broth (Sigma Aldrich) overnight at 37 °C on a rotary shaker operating at 125 rpm^51^. The culture was subsequently centrifuged at 200 rpm for 2 min and the supernatant was removed in order to transfer the bacterial cells into sterile DMEM Glutamax medium with FBS and without any antibiotics and without Caco-2 cells. The bacterial cells were incubated in this medium for at least 30 min at room temperature. Bacteria concentration of this medium was found as 3.8 · 10^8^ CFU ml^−1^ by inoculating the serial dilutions of the medium on LB agar plates overnight.

In order to study cell-bacteria interactions, intestinal bacteria, *E. coli*, were injected (1.9 · 10^7^ cells ml^−1^) together with the culture medium into the microchip starting on the 8^th^ day of culture for 36 h. The experiments were performed under both fluid flow and static conditions to provide a comparative study. The microchannels at each side of the hydrogel compartments were used to introduce bacterial cells into the microchip. On the last day, culture medium was switched to antibiotic-free culture medium in both microchips. Two microchips (one was treated under static, the other was treated under fluidic conditions) were then filled with the above-mentioned bacteria-including medium mixture and incubated for 36 h. A control study was performed in a second set of microchips (one was treated under static, the other was treated under flow conditions) that were incubated without the bacteria-culture medium mixture and incubated for 36 h. Live/dead staining was applied to screen the survival of Caco-2 cells using calcein-AM and ethidium homodimer-1, respectively.

For the preliminary drug screening study, GFP expressing *E. coli* [pRSETB] were incubated for 1.5 h in a microchip containing 8-days cultured Caco-2 cells. The microchannels were then carefully washed with antibiotic-free cell culture medium for 1.5 h to remove non-adherent *E. coli* cells. Finally, a chloramphenicol supplemented (34 µg ml^−1^) culture medium was pumped into the microchip at 300 µl h^−1^ flow rate for 36 h in order to remove the adherent *E. coli* cells from Caco-2 cells under 37 °C and 5% CO_2_ culture conditions.

### Morphological analysis

Phase contrast images were recorded throughout experiments using an EVOS FL imaging system (ThermoFisher Scientific) equipped with EVOS phase contrast objectives, and GFP and DAPI filter cubes. For the fluorescence staining, F-actin and nuclei of the cells were stained in the Caco-2 cells after fixation in 4% (v/v) paraformaldehyde and permeabilization in 0.3% (v/v) Triton X-100 (Sigma Aldrich) using ActinGreen™ 488 (ThermoFisher Scientific, USA) and NucBlue (ThermoFisher Scientific, USA). After the staining process, the cells were scanned using the EVOS FL imaging system and a laser scanning confocal microscope (Zeiss LSM 510, Germany).

## Electronic supplementary material

Supplementary information file

Supplementary information movie S1
