# Supplementary material for: Compartmentalized 3D Tissue Culture Arrays under Controlled Microfluidic Delivery
Source: Sci Rep. 2017 Jun 13;7:3381. doi: 10.1038/s41598-017-01944-5 (PMC5469754; doi:10.1038/s41598-017-01944-5)
Supplement: Supplementary file 1 — Supplementary information file [file 41598_2017_1944_MOESM1_ESM.pdf]

Supplementary Information

Compartmentalized 3D Tissue Culture Arrays under Controlled Microfluidic Delivery

Burcu Gumuscu[a,*], Hugo J. Albers[a,b], Albert van den Berg[a], Jan C. T. Eijkel[a], and Andries D. van der Meer[b,*]

[a]BIOS Lab-on-a-Chip Group, MESA+ Institute for Nanotechnology, MIRA Institute for Biomedical Technology and Technical Medicine, University of Twente, 7500AE, Enschede, The Netherlands
[b]Applied Stem Cell Technologies Group, MIRA Institute for Biomedical Technology and Technical Medicine, University of Twente, 7500AE, Enschede, The Netherlands

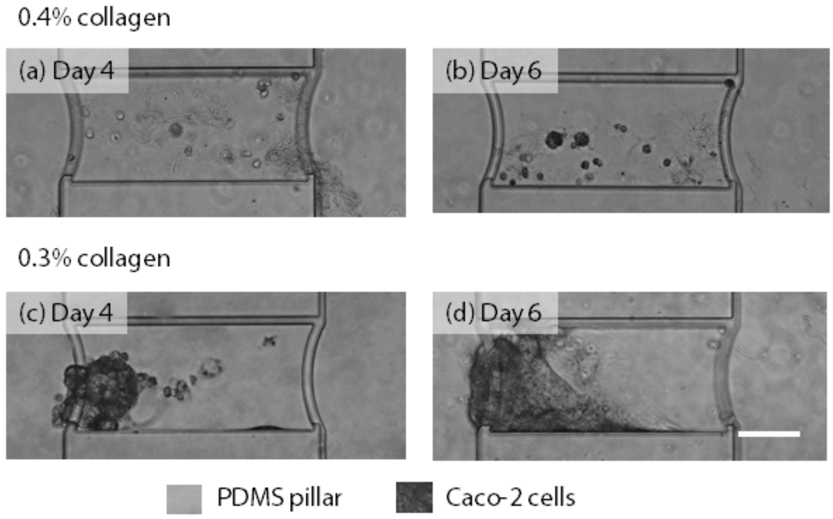

Figure S1. Top-view phase contrast microscopy images of Caco-2 cells cultured in **(a-b)** 0.4% **(c-d)** and 0.3% collagen concentrations under 300 µl h^{-1} flow rate. Scale bar is 125 µm.

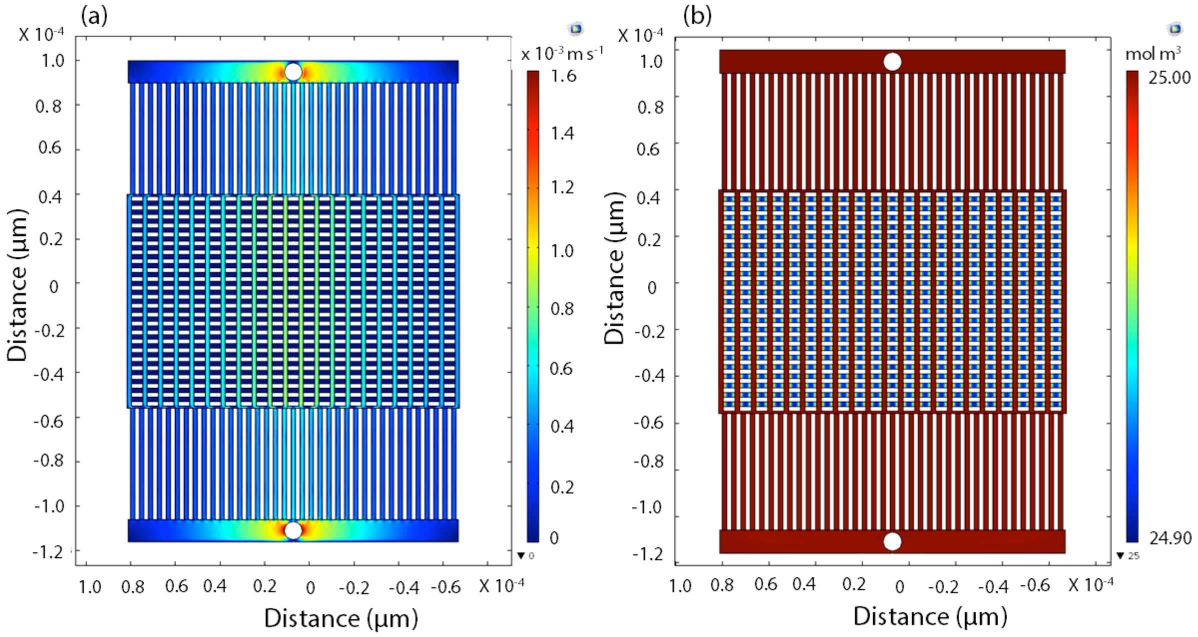

Figure S2. **(a)** Laminar flow simulation in the microchip. The flow velocity is the highest in the center of the rectangular chamber and gradually decreases towards the sides. **(b)** Predicted concentration distribution of glucose throughout the microchip assuming a glucose consumption in the compartments. The scale bar presents the same value from top to bottom. Please note from the scale bar that the glucose concentration is practically uniform.

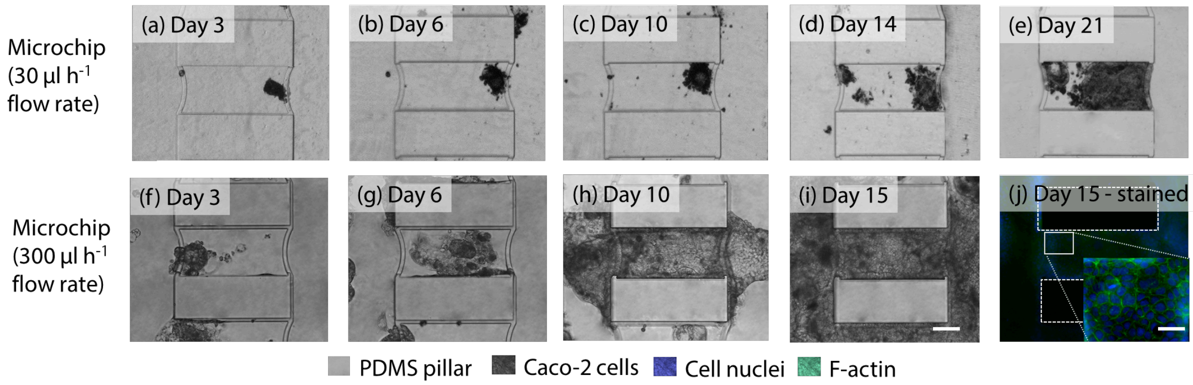

Figure S3. Top-view phase contrast images of long-term Caco-2 cultures in the microchip. The results are shown for the microchip perfused under **(a-e)** 30 µl h^{-1}, and **(f-j)** 300 µl h^{-1} flow rates. In the fluorescence images (bottom line, day 15) staining was applied: blue colors present cell nuclei (NucBlue), and green colors present cytoskeleton (F-actin). Scale bar for the images is 125 µm. The scale bar in the close-up image is 40 µm.

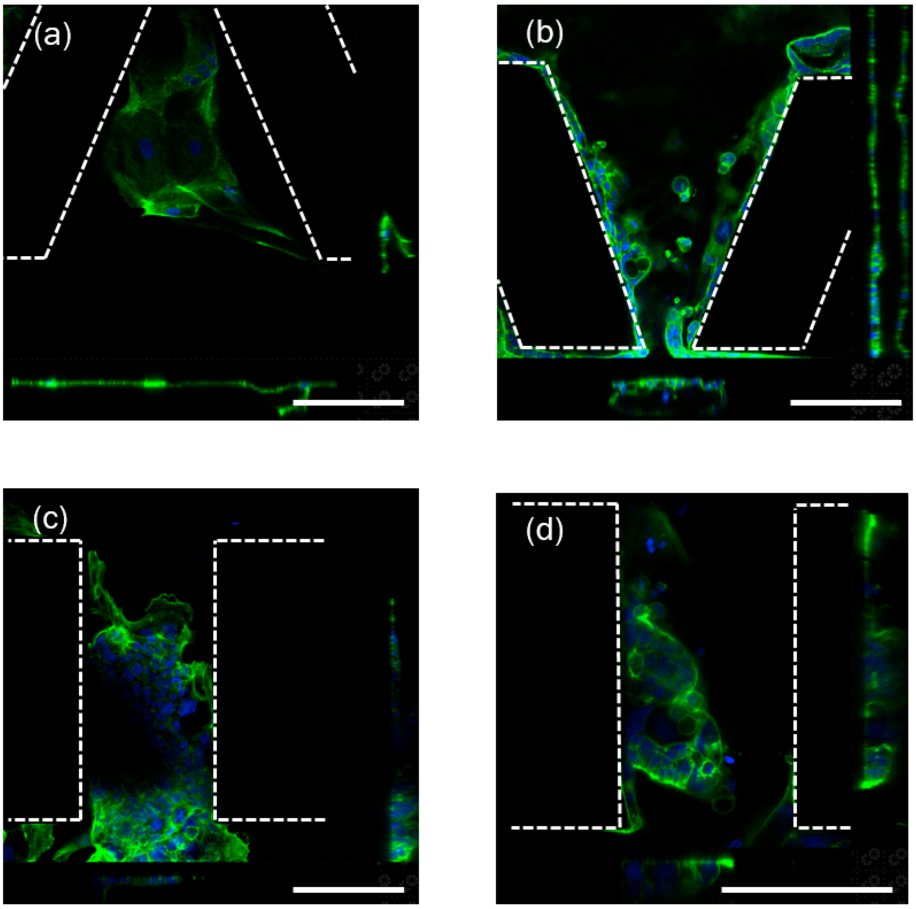

Fig. S4. Confocal fluorescence microscopy images of Caco-2 cells. Top-down and orthogonal views are portrayed in each image. **(a, b)** Triangular compartments and **(c, d)** rectangular compartments of Caco-2 cells cultured under 300 µl h^{-1} flow rate of culture medium. DAPI and GFP represent cell nuclei and F-actin filaments, respectively. Cells form tubes and irregular 3D folds. Scale bars are 200 µm.

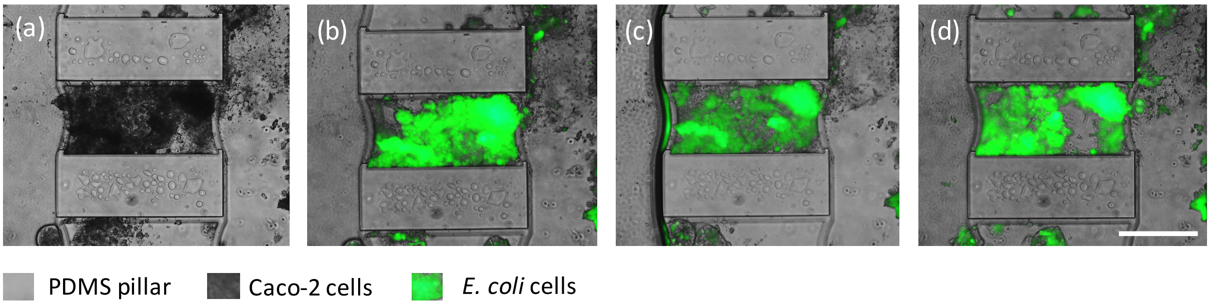

Figure S5. Overlay of phase contrast and fluorescent microscopy images of a GFP-expressing E. coli and Caco-2 co-culture followed by a chloramphenicol treatment **(a)** before incubation with GFP-expressing *E. coli*. **(b)** after a 1.5 h incubation of GFP-expressing *E. coli*. **(c)** after 30 min chloramphenicol treatment **(d)** after 36 h chloramphenicol treatment. Scale bar is 250 µm.

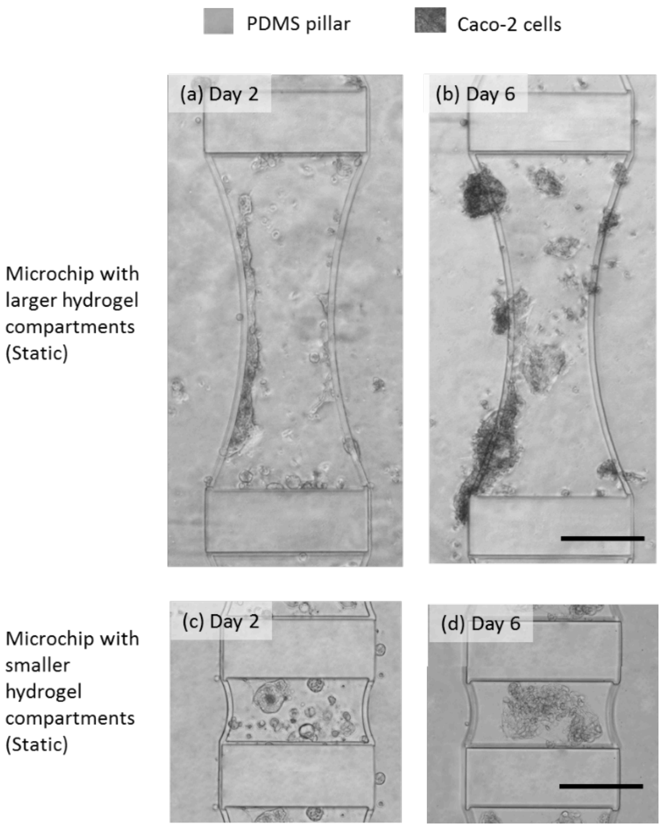

Figure S6. Top-view phase contrast microscopy images of Caco-2 cells cultured under static conditions in compartments with various sizes including **(a, b)** larger and **(c, d)** smaller compartment designs. Scale bars are 125 µm.

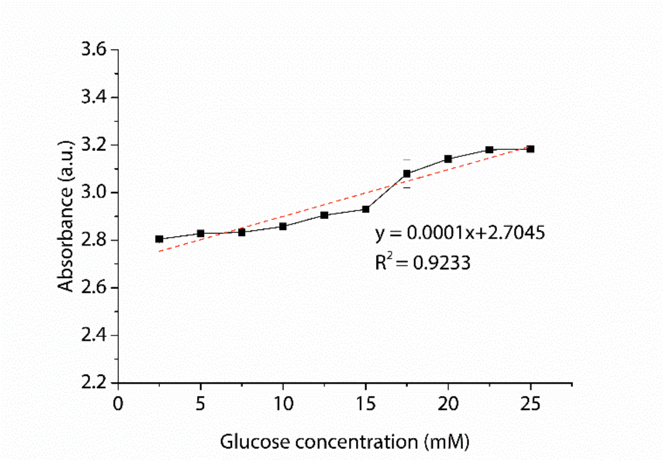

Figure S7. Calibration curve used in glucose concentration measurements.

Movie S1. Rotating confocal image of Caco-2 cells grown in a rectangular shaped compartment on day 8. Cells formed tube structures with 3D folds and podia inside. In the fluorescence images (bottom line, day 15) staining was applied: blue colors present cell nuclei (NucBlue), and green colors present cytoskeleton (F-actin).
